# Topical blood products modulate the effects of ophthalmic antibiotics against common bacterial pathogens in dogs with infectious keratitis

**DOI:** 10.3389/fvets.2024.1417842

**Published:** 2024-07-12

**Authors:** Melissa A. Kubai, Mackenzie M. Roy, Chloe C. Stinman, Danielle E. Kenne, Rachel A. Allbaugh, Lionel Sebbag

**Affiliations:** ^1^Department of Veterinary Clinical Sciences, Iowa State University, College of Veterinary Medicine, Ames, IA, United States; ^2^Veterinary Diagnostic Laboratory, Iowa State University, College of Veterinary Medicine, Ames, IA, United States; ^3^Koret School of Veterinary Medicine, Hebrew University of Jerusalem, Rehovot, Israel

**Keywords:** antimicrobial susceptibility testing, antibiotic resistance, bacterial keratitis, blood derivatives, minimal inhibitory concentrations, protein binding

## Abstract

Bacterial keratitis is a common and serious condition that often leads to vision impairment and potential loss of the eye if not treated promptly and adequately. Topical blood products are often used concurrently with topical antibiotics, helping to mitigate corneal ‘melt’ from proteases released on the ocular surface. However, blood products are rich in albumin and could affect the efficacy of antibiotics due to drug-protein binding. In this study, serum and plasma samples were harvested from 10 healthy dogs and 10 healthy horses, obtaining fresh and frozen (1 month at −20°C) aliquots for *in vitro* experiments. Albumin levels were quantified using species-specific ELISA kits. Thirty bacteria (10 *Staphylococcus pseudintermedius*, 10 *Streptococcus canis*, 10 *Pseudomonas aeruginosa*), isolated from canine patients with infectious keratitis, were each tested with blank plates as well as commercial susceptibility plates (Sensititre™ JOEYE2) to assess the minimal inhibitory concentration (MIC) of 17 different antibiotics in the absence (control) or presence of eight test groups: serum or plasma (fresh or frozen) from canines or equines. Albumin concentrations ranged from 13.8–14.6 mg/mL and 25.9–26.5 mg/mL in canine and equine blood products, respectively. A direct antimicrobial effect was observed mostly with equine vs. canine blood products (specifically serum and to a lesser degree plasma), and mostly for *Staphylococcus pseudintermedius* isolates. MICs generally increased in the presence of blood products (up to 10.8-fold), although MICs also decreased (down to 0.25-fold) for selected antibiotics and ocular pathogens. Median (range) fold changes in MICs were significantly greater (*p* = 0.004) with the canine blood products [2 (0.67–8.1)] than the equine blood products [2 (0.5–5)]. In practice, clinicians should consider equine over canine blood products (lesser impact on antimicrobial susceptibility), serum over plasma (greater antimicrobial effects), and administering the blood product ≥15 min following the last antibiotic eyedrop to minimize the amount of albumin-antibiotic binding in tear film.

## 1 Introduction

Bacterial keratitis is a major global cause of avoidable visual impairment and ocular discomfort in many species, including humans, dogs, cats, and horses (1–4). Following an injury to the eye (e.g., trauma, foreign body), corneal wounds have a high tendency towards infection given the presence of indigenous microflora on the ocular surface that can become pathogenic in diseased eyes. Despite having appropriate antibacterial treatments for most of the pathogens implicated in bacterial keratitis, clinical outcomes are often poor due to devastating effects such as corneal ‘melt’ (keratomalacia), scarring, perforation, loss of vision, or loss of the eye.

Medical management of bacterial keratitis is complex and generally involves different therapeutics. In particular, blood products such as serum or plasma are often used concurrently with topical antibiotics to promote corneal wound healing (1). On one hand, serum or plasma can mitigate corneal collagenolysis (‘melt’) by inhibiting endogenous matrix metalloproteinases (MMPs) released by bacteria and inflammatory cells (5, 6). On the other hand, these blood products are rich in albumin (7), a protein that was recently shown to reduce the efficacy of common ophthalmic antibiotics as only the unbound portion of an antibiotic is microbiologically active (8, 9). Indeed, Sebbag et al. (10) showed that serum albumin increased the minimal inhibitory concentrations (MICs) of 17 different antibiotics in a dose-dependent, bacteria-specific, and antibiotic-specific manner.

The main goal of this study was to evaluate the potential impact of two blood products (i.e., serum, plasma) on the efficacy of ophthalmic antibiotics against common ocular pathogens isolated from patients with bacterial keratitis. A secondary objective was to compare blood products from two species (i.e., dogs, horses) and two storage conditions (i.e., fresh, frozen) in order to mimic the clinical use of blood products in general ophthalmology practice.

## 2 Materials and methods

### 2.1 Serum and plasma

#### 2.1.1 Collection

The study was reviewed and approved by the Institutional Animal Care and Use Committee at Iowa State University (protocol # 21–197). Ten healthy Beagles and ten healthy horses were recruited from the teaching colony at Iowa State University, College of Veterinary Medicine. Animals were excluded if a topical or systemic antibiotic was administered in the last 30 days. Each animal underwent two sessions of blood collection separated by 1 month, freezing the blood products at −20°C following for the first session (frozen samples) and refrigerating the blood products at 4°C for <24 h following the second session (fresh samples) until the *in vitro* experiments (see below). Briefly, 30 mL of blood was collected via venipuncture from each animal at each session, splitting the collected blood into plain red-top tubes or heparinized green-top tubes to obtain serum or plasma following tube centrifugation, respectively.

#### 2.1.2 Albumin quantification

Albumin levels were quantified in each sample (i.e., fresh or frozen; serum or plasma; canine or equine) using species-specific ELISA kits from LifeSpan Biosciences Inc. (Canine LS-F8537; Equine LS-F10158) according to the manufacturer’s instructions.

### 2.2 Antimicrobial susceptibility testing

Thirty bacterial isolates were examined, representative of the three most common bacterial species identified in canine patients with ulcerative keratitis, that is, *Staphylococcus pseudintermedius* (*n* = 10), *Streptococcus canis* (*n* = 10), and *Pseudomonas aeruginosa* (*n* = 10) (11). Bacterial isolates cultured from canine patients were revived from the −80°C freezer by thawing at room temperature and grown on tryptic soy agar with 5% sheep blood agar plates. All plates were incubated at 35 ± 2°C with 5–10% CO_2_ for a total time of 24–48 h. Each bacterial isolate was then diluted to 1.5 × 10^8^ colony forming units per milliliter (CFU/mL) in sterile physiological saline, confirmed to be pure by instilling and incubating the solution onto blood agar plates.

**Figure 1:**
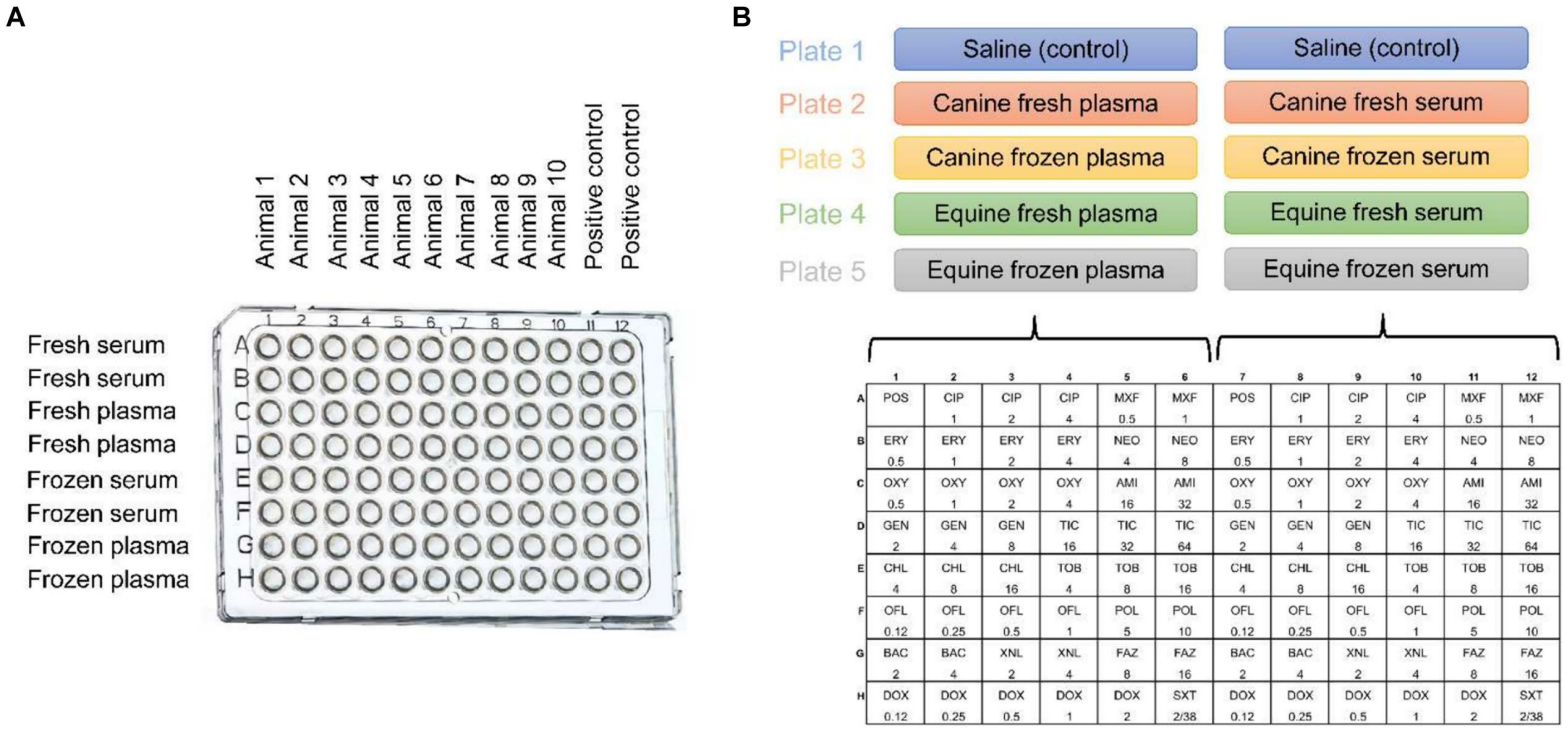
Graphical representation of the study design involving 30 bacteria isolates from canine patients with infectious keratitis (10 *Staphylococcus pseudintermedius*, 10 *Streptococcus canis*, 10 *Pseudomonas aeruginosa*) and blood products (serum or plasma, fresh or frozen) retrieved from healthy dogs and horses. **(A)** Two blank plates per bacterial isolate, with wells containing the bacterial broth and canine (plate 1) or equine (plate 2) blood products, but no antibiotics; Animal = dog or horse. **(B)** Five Sensititre™ JOEYE2 plates per bacterial isolate, with wells containing the bacterial broth, saline (control) or canine/equine blood products, and one of 17 different antibiotics at various concentrations (μg/ml).

Eight blood products were tested in the study, namely: Canine serum fresh (cS-Fsh), canine plasma fresh (cP-Fsh), canine serum frozen (cS-Frz), canine plasma frozen (cP-Frz), equine serum fresh (eS-Fsh), equine plasma fresh (eP-Fsh), equine serum frozen (eS-Frz), equine plasma frozen (eP-Frz). Two sets of experiments were performed (Figure 1):

(1) *Control plate*: Two blank plates (Corning 96-well Clear Polystyrene Microplates, Corning Inc.) with no antibiotics were used for each bacterial isolate, one for the blood products of the ten dogs and one for the blood products of the ten horses. Bacterial broth was manually pipetted into all wells along with 25-μl of blood product in duplicates for columns 1 to 10 (Canine plate: cS-Fsh, cP-Fsh, cS-Frz, cP-Frz; Equine plate: eS-Fsh, eP-Fsh, eS-Frz, eP-Frz) and 25-μl of sterile saline for columns 11 and 12 (positive control).
(2) *Antimicrobial susceptibility plates*: Each bacterial broth was transferred into glass tubes containing an equal volume of sterile saline (control) or blood product (cS-Fsh, cP-Fsh, cS-Frz, cP-Frz, eS-Fsh, eP-Fsh, eS-Frz, eP-Frz). Then, an automated inoculation delivery system (Sensititre AIM™, Thermo Scientific Inc.) was used to transfer each broth/blood product solution onto standard plates (50-μL in each of the 48 wells) that are specifically manufactured for sensitivity testing in ophthalmology (Sensititre™ JOEYE2 plate, Thermo Scientific Inc.). These plates are preloaded with serial dilutions of 17 different antibiotics that are routinely used in veterinary patients to manage bacterial keratitis, including examples of the following common topical antibiotics: bacitracin, tobramycin, erythromycin, oxytetracycline, gentamicin, chloramphenicol, cefazolin, neomycin and ofloxacin.^1^ A total of 5 JOEYE2 plates were used for each bacterial isolate (Figure 1). Following CLSI guidelines, all positive control plates and Sensititre™ plates were incubated at 35°C ± 2°C for either 16-20 h (*Pseudomonas* sp.), 20-24 h (*Streptococcus* sp.), or 24 h (*Staphylococcus* sp.), followed by data recording using a digital MIC viewing system (Sensititre Vizion™, Thermo Scientific Inc.). The presence or absence of bacterial growth was recorded in each well of the blank plates. For each of the 17 antibiotics in JOEYE2 plates, the MIC was read as the lowest concentration of antimicrobial agent that completely inhibited organism growth, a result that was accompanied by a clinical interpretation (i.e., susceptible, intermediate, resistant, or non-interpretable) based on the breakpoints described in the VET08 and M100 CLSI documents (12, 13).

### 2.3 Data analysis

Normality of data was assessed using the Shapiro–Wilk test. Mann–Whitney tests were used to compare albumin levels of each blood product between dogs and horses, while paired t tests were used to compare albumin levels between serum vs. plasma and fresh vs. frozen samples. For each antibiotic (*n* = 17) and bacterial species (*Staphylococcus pseudintermedius*, *Streptococcus canis*, *Pseudomonas aeruginosa*), MICs obtained with blood product were compared to control (sterile saline) using the Kruskal-Wallis test and post-hoc Dunnett’s method. The Mann–Whitney test was used to compare the overall change in MICs (blood products vs. control) between canine and equine blood products. Statistical analyses were performed with SigmaPlot 14.5 (Systat software, Point Richmond, CA), and *p* values <0.05 were considered significant.

## 3 Results

### 3.1 Albumin levels

**Figure 2:**
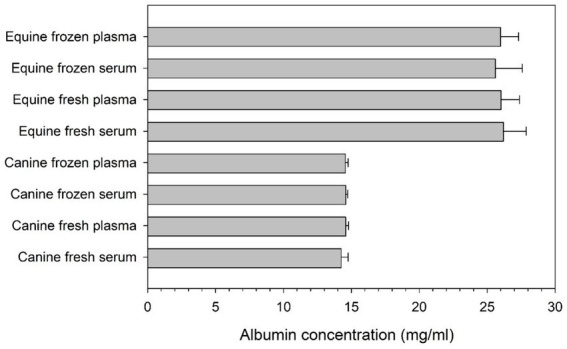
Bar chart depicting the mean + standard deviation concentration of albumin (mg/ml) in serum and plasma of 10 healthy dogs and 10 healthy horses, as quantified in fresh and frozen samples.

Albumin levels in canine and equine blood products, fresh or frozen, are summarized in Figure 2. Albumin concentrations were significantly higher (*p* < 0.001) in equine blood products (range 25.9–26.5 mg/mL) compared to canine blood products (range 13.8–14.6 mg/mL). However, no statistical differences were noted between serum and plasma albumin levels (*p* ≥ 0.086) or between fresh and frozen samples (*p* ≥ 0.058) in any species.

### 3.2 Control plates

Bacterial growth was observed in all positive control wells. However, selected wells receiving blood product (but no antibiotics) had negative bacterial growth, a finding that was unexpected and more common with equine vs. canine blood products (Table 1). For instance, growth of *Staphylococcus pseudintermedius* was inhibited in 165/200 (82.5%) and 0/200 (0%) of the wells that received equine fresh serum and canine fresh serum, respectively.

**Table 1:** Number (percent) of wells without bacterial growth, following exposure of bacterial isolates (*n* = 10 *Staphylococcus pseudintermedius*, *n* = 10 *Streptococcus canis*, *n* = 10 *Pseudomonas aeruginosa*) to blood products obtained from 10 healthy dogs and 10 healthy horses.

	Canine				Equine			
	Fresh serum	Fresh plasma	Frozen serum	Frozen plasma	Fresh serum	Fresh plasma	Frozen serum	Frozen plasma
*Staphylococcus pseudintermedius*	0/200 (0%)	0/200 (0%)	0/200 (0%)	0/200 (0%)	165/200 (82.5%)	15/200 (7.5%)	195/200 (97.5%)	2/200 (1%)
*Streptococcus canis*	0/200 (0%)	0/200 (0%)	0/200 (0%)	0/200 (0%)	0/200 (0%)	0/200 (0%)	0/200 (0%)	0/200 (0%)
*Pseudomonas aeruginosa*	30/200 (15%)	30/200 (15%)	0/200 (0%)	0/200 (0%)	91/200 (45.5%)	70/200 (35%)	68/200 (34%)	92/200 (46%)

The canine and equine blood products were either serum or plasma that were either fresh or frozen.

### 3.3 Antimicrobial susceptibility testing

**Figure 3:**
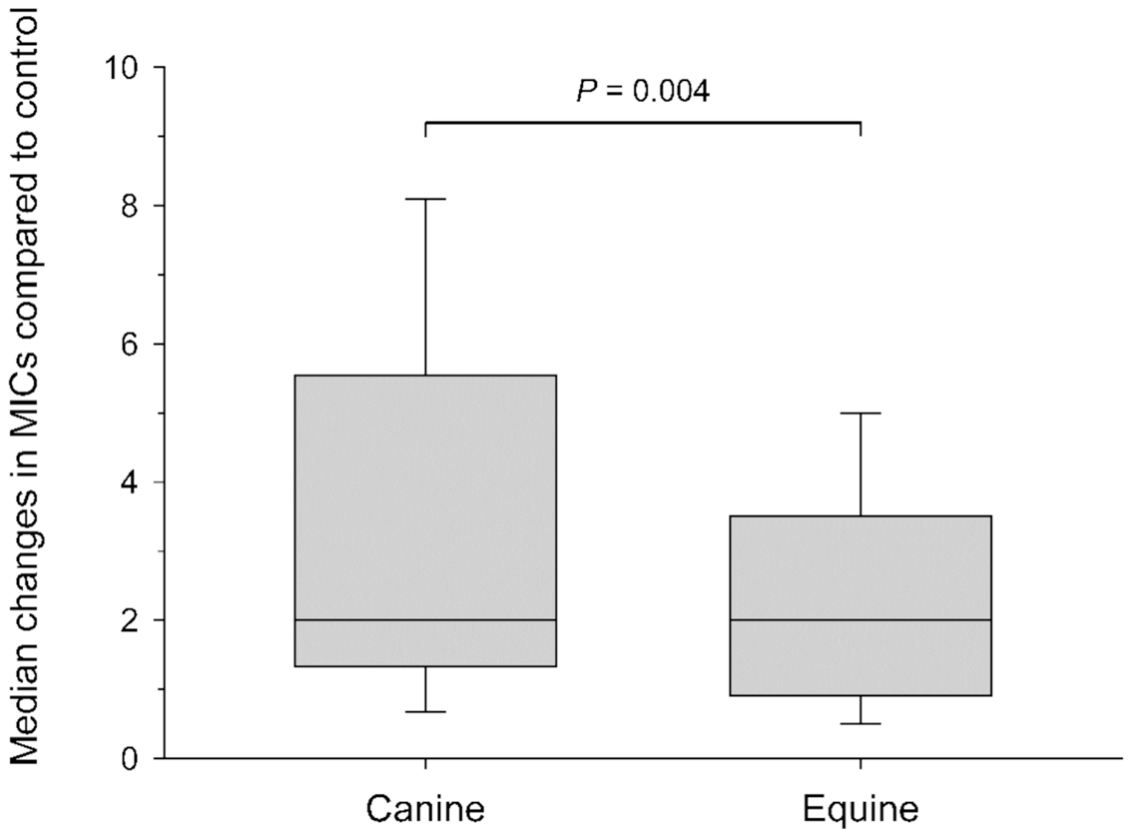
Box-and-whisker plots depicting changes in minimal inhibitory concentrations (MICs) when comparing control (saline) to blood products (plasma and serum; fresh and frozen) for all bacterial isolates combined. Median values are shown by a horizontal line. First and third quartiles (25th and 75th percentiles) are represented by the lower and upper limits of the box, respectively. The 2.5th and the 97.5th percentiles are shown as the lower and upper whiskers, respectively.

Table 2 depicts a representative example of one *Staphylococcus pseudintermedius* isolate, showing the differences in MICs when compared to control and associated changes in clinical interpretation (i.e., *sensitive* to *intermediate*, *intermediate* to *resistant*, *sensitive* to *resistant*); for instance, the clinical interpretation for tobramycin changed from *sensitive* to *intermediate* with the addition of each canine blood product (cS-Fsh, cP-Fsh, cS-Fzn, cP-Fzn), and from *sensitive* to *resistant* with the addition of eS-Fsh or eP-Fzn. For each bacterial species (n = 10 isolates), results are summarized in Table 3 (*Staphylococcus pseudintermedius*), Table 4 (*Streptococcus canis*), and Table 5 (*Pseudomonas aeruginosa*). Median MICs generally increased with the addition of blood product to the growth media (up to 10.81-fold), but sometimes the MIC decreased (down to 0.25-fold). When results were grouped for all blood products and all bacterial isolates, median (range) fold changes in MICs were significantly greater (*p* = 0.004) with the canine blood products [2 (0.67–8.1)] compared to the equine blood products [2 (0.5–5)] (Figure 3).

**Table 2:** Changes in minimal inhibitory concentrations (MICs, in μg/ml) and associated clinical interpretation when one representative bacterial isolate of *Staphylococcus pseudintermedius* was exposed to 17 different ophthalmic antibiotics and firstly saline (control) then eight different blood products originating from healthy dogs and horses.

	Canine fresh plasma	Canine fresh serum	Canine frozen plasma	Canine frozen serum	Equine fresh plasma	Equine fresh serum	Equine frozen plasma	Equine frozen serum
Amikacin	16→16 NI→NI	16→16 NI→NI	16→16 NI→NI	16→16 NI→NI	16→16 NI→NI	16→16 NI→NI	16→16 NI→NI	16→16 NI→NI
Bacitracin	4→4 NI→NI	4→4 NI→NI	4→4 NI→NI	4→4 NI→NI	4→4 NI→NI	4→2 (0.5) NI→NI	4→4 NI→NI	4→2 (0.5) NI→NI
Cefazolin	8→8 S→S	8→16 (2) S→R	8→8 S→S	8→8 S→S	8→8 S→S	8→8 S→S	8→8 S→S	8→8 S→S
Ceftiofur	2→4 (2) NI→NI	2→4 (2) NI→NI	2→4 (2) NI→NI	2→4 (2) NI→NI	2→2 NI→NI	2→2 NI→NI	2→4 (2) NI→NI	2→2 NI→NI
Chloramphenicol	4→8 (2) S→S	4→4 S→S	4→8 (2) S→S	4→16 (4) S→R	4→4 S→S	4→4 S→S	4→4 S→S	4→4 S→S
Ciprofloxacin	1→4 (4) S→R	1→1 S→S	1→1 S→S	1→1 S→S	1→4 (4) S→R	1→1 S→S	1→1 S→S	1→1 S→S
Doxycycline	0.5→0.5 S→S	0.5→1 (2) S→S	0.5→0.5 S→S	0.5→1 (2) S→S	0.5→0.25 (0.5) S→S	0.5→0.25 (0.5) S→S	0.5→0.25 (0.5) S→S	0.5→0.25 (0.5) S→S
Erythromycin	0.5→4 (8) S→I	0.5→4 (8) S→I	0.5→4 (8) S→I	0.5→4 (8) S→I	0.5→4 (8) S→I	0.5→0.5 S→S	0.5→4 (8) S→I	0.5→0.5 S→S
Gentamicin	2→4 (2) S→I	2→8 (4) S→R	2→8 (4) S→R	2→8 (4) S→R	2→8 (4) S→R	2→2 S→S	2→8 (4) S→R	2→2 S→S
Moxifloxacin	0.5→1 (2) S→I	0.5→0.5 S→S	0.5→0.5 S→S	0.5→0.5 S→S	0.5→1 (2) S→I	0.5→0.5 S→S	0.5→0.5 S→S	0.5→0.5 S→S
Neomycin	4→8 (2) S→I	4→8 (2) S→I	4→8 (2) S→I	4→8 (2) S→I	4→4 S→S	4→4 S→S	4→4 S→S	4→8 (2) S→I
Ofloxacin	0.5→1 (2) S→I	0.5→1 (2) S→I	0.5→1 (2) S→I	0.5→1 (2) S→I	0.5→0.25 (0.5) S→S	0.5→1 (2) S→I	0.5→1 (2) S→I	0.5→0.12 (0.25) S→S
Oxytetracycline	0.5→4 (8) S→R	0.5→1 (2) S→S	0.5→0.5 S→S	0.5→0.5 S→S	0.5→4 (8) S→R	0.5→0.5 S→S	0.5→0.5 S→S	0.5→2 (4) S→S
Polymyxin B	10→10 NI→NI	10→10 NI→NI	10→10 NI→NI	10→10 NI→NI	10→5 (0.5) NI→NI	10→5 (0.5) NI→NI	10→10 NI→NI	10→5 (0.5) NI→NI
Ticarcillin	16→16 S→S	16→16 S→S	16→16 S→S	16→16 S→S	16→64 (4) S→R	16→16 S→S	16→16 S→S	16→16 S→S
Tobramycin	4→8 (2) S→I	4→8 (2) S→I	4→8 (2) S→I	4→8 (2) S→I	4→4 S→S	4→16 (4) S→R	4→16 (4) S→R	4→4 S→S
Trimethoprim	2→2 S→S	2→2 S→S	2→2 S→S	2→2 S→S	2→2 S→S	2→2 S→S	2→2 S→S	2→2 S→S

Boxes highlighted in red or blue represent an increase or decrease in MIC when compared to saline control, respectively. S, sensitive; I, intermediate; R, resistant; NI, not interpretable.

**Table 3:** Changes in minimal inhibitory concentrations (MICs) of ophthalmic antibiotics used against *Staphylococcus pseudintermedius* isolated from canine patients with infectious keratitis.

	Canine fresh plasma	Canine fresh serum	Canine frozen plasma	Canine frozen serum	Equine fresh plasma	Equine fresh serum	Equine frozen plasma	Equine frozen serum	*p* value
Amikacin	–	–	–	–	–	–	–	–	1.000
Bacitracin	–	–	–	–	–	–	–	0.75	<0.001
Cefazolin	–	–	–	–	–	–	–	–	0.433
Ceftiofur	–	–	–	–	–	–	–	–	0.181
Chloramphenicol	1.5	1.5	**2**	**2**	–	–	–	–	<0.001
Ciprofloxacin	2.5	–	1.5	1.5	–	–	–	–	0.302
Doxycycline	2.5	2	2.5	2	1.5	0.5	1.5	0.5	0.072
Erythromycin	8	4.5	8	4.5	1	–	–	–	0.136
Gentamicin	3	3	4	3	–	–	–	–	0.006
Moxifloxacin	–	–	–	–	–	–	–	–	0.686
Neomycin	1.5	–	–	–	1.5	–	1.5	–	0.176
Ofloxacin	4	4	3	4	4	2	3	0.48	<0.001
Oxytetracycline	8	2	5	4.5	5	–	5	2.5	0.747
Polymyxin B	–	–	–	–	–	0.5	–	0.75	<0.001
Ticarcillin	–	–	–	–	–	–	–	–	0.134
Tobramycin	–	–	–	–	–	–	–	–	0.189
Trimethoprim	–	–	–	–	–	–	–	–	1.000

Results are depicted as fold change in median MIC (*n* = 10 *Streptococcus canis* pseudintermedius isolates) when comparing standard test medium (i.e., no blood product = control) vs. test medium supplemented with canine or equine blood products (serum or plasma; fresh or frozen). – indicates no change in MIC. Boxes highlighted in red or blue represent an increase or decrease in MIC when compared to control, respectively. *P* values represent statistical comparisons among all groups (Kruskal-Wallis test). Bolded text indicates statistical differences of a specific blood product vs. control (post-hoc Dunnett’s).

**Table 4:** Changes in minimal inhibitory concentrations (MICs) of ophthalmic antibiotics used against *Streptococcus canis* isolated from canine patients with infectious keratitis.

	Canine fresh plasma	Canine fresh serum	Canine frozen plasma	Canine frozen serum	Equine fresh plasma	Equine fresh serum	Equine frozen plasma	Equine frozen serum	*p* value
Amikacin	–	–	–	–	–	–	–	–	1.000
Bacitracin	2	2	2	2	2	2	2	2	<0.001
Cefazolin	–	–	–	–	–	–	–	–	0.528
Ceftiofur	–	–	–	–	–	–	–	–	0.437
Chloramphenicol	–	–	–	–	–	–	–	–	0.768
Ciprofloxacin	–	–	–	–	–	–	–	–	0.433
Doxycycline	8.11	8.11	10.81	5.41	2.70	2.70	2.70	5.41	0.012
Erythromycin	–	–	–	–	–	–	–	–	0.632
Gentamicin	**2**	**2**	**2**	**2**	**2**	**2**	**2**	**2**	< 0.001
Moxifloxacin	–	–	–	–	–	–	–	–	0.433
Neomycin	–	–	–	–	–	–	–	–	1.000
Ofloxacin	**2**	**2**	**2**	**2**	**2**	**2**	**2**	**2**	< 0.001
Oxytetracycline	2	2	2	2	2	2	2	2	0.005
Polymyxin B	–	–	–	–	–	–	–	–	0.433
Ticarcillin	–	–	–	–	–	–	–	–	1.000
Tobramycin	**2**	**2**	**2**	**2**	**2**	**2**	**2**	**2**	< 0.001
Trimethoprim	–	–	–	–	–	–	–	–	1.000

Results are depicted as fold change in median MIC (*n* = 10 *Streptococcus canis* isolates) when comparing standard test medium (i.e., no blood product = control) vs. test medium supplemented with canine or equine blood products (serum or plasma; fresh or frozen). – indicates no change in MIC. Boxes highlighted in red or blue represent an increase or decrease in MIC when compared to control, respectively. *P* values represent statistical comparisons among all groups (Kruskal-Wallis test). Bolded text indicates statistical differences of a specific blood product vs. control (post-hoc Dunnett’s).

**Table 5:** Changes in minimal inhibitory concentrations (MICs) of ophthalmic antibiotics used against *Pseudomonas aeruginosa* isolated from canine patients with infectious keratitis.

	Canine fresh plasma	Canine fresh serum	Canine frozen plasma	Canine frozen serum	Equine fresh plasma	Equine fresh serum	Equine frozen plasma	Equine frozen serum	*p* value
Amikacin	–	–	–	–	–	–	–	–	0.438
Bacitracin	–	–	–	–	–	–	–	–	0.05
Cefazolin	–	–	–	–	–	–	–	–	0.122
Ceftiofur	–	–	–	–	–	–	–	–	0.014
Chloramphenicol	**0.5**	–	–	–	–	**0.5**	**0.25**	–	<0.001
Ciprofloxacin	–	–	–	–	–	–	–	–	0.528
Doxycycline	–	–	–	–	–	–	–	–	0.098
Erythromycin	–	–	–	–	–	–	–	–	0.188
Gentamicin	1.33	1.33	1.33	1.33	0.67	1.33	0.67	0.67	0.002
Moxifloxacin	0.67	1.33	0.67	1.33	1.33	0.67	0.67	0.67	0.045
Neomycin	**2**	2	**2**	**2**	2	**2**	2	2	< 0.001
Ofloxacin	–	–	–	–	–	1.5	0.5	–	0.033
Oxytetracycline	2	2	2	2	2	1	2	2	< 0.001
Polymyxin B	–	–	–	–	–	–	–	–	0.277
Ticarcillin	–	–	–	–	–	–	–	–	0.232
Tobramycin	–	–	–	–	–	–	–	–	1.000
Trimethoprim	–	–	–	–	–	–	–	–	1.000

Results are depicted as fold change in median MIC (*n* = 10 *Pseudomonas aeruginosa* isolates) when comparing standard test medium (i.e., no blood product = control) vs. test medium supplemented with canine or equine blood products (serum or plasma; fresh or frozen). – indicates no change in MIC. Boxes highlighted in red or blue represent an increase or decrease in MIC when compared to control, respectively. *P* values represent statistical comparisons among all groups (Kruskal-Wallis test). Bolded text indicates statistical differences of a specific blood product vs. control (post-hoc Dunnett’s).

## 4 Discussion

Blood products modulated the MICs of ophthalmic antibiotics against common ocular pathogens from patients with bacterial keratitis, an effect that varied with the specific bacterial species, antibiotic, type of blood product (serum or plasma), storage condition (fresh or frozen) and species of origin (canine or equine). MICs generally increased in the presence of blood products (up to 10.8-fold), although MICs also decreased (down to 0.25-fold) for selected antibiotics and ocular pathogens.

Elevations in MICs noted in the present study are likely related (at least in part) to the presence of albumin at high concentrations in serum and plasma of dogs and horses. Here, albumin levels quantified in canine (13.8–14.6 mg/mL) and equine blood products (25.9–26.5 mg/mL) were much higher than the minimum albumin level (0.5 mg/mL) shown to impact the efficacy of selected ophthalmic antibiotics against common ocular pathogens (10). In fact, albumin levels in blood products were overall higher than the ones quantified on the ocular surface of dogs with spontaneously occurring breakdown of the blood-tear barrier (10, 14). In other words, if a topical antibiotic mixes with a topical blood product on the ocular surface, a fraction of the antibiotic becomes albumin-bound and is rendered inactive (8, 9), thereby increasing the amount of antibiotic required to inhibit bacterial growth. Clinically, increased MICs from blood products may translate into poorer clinical outcomes. Indeed, a recent study by our research group showed that bacterial keratitis deteriorated in selected canine patients despite aggressive antibiotic therapy (10), while previous reports in human patients showed slower corneal wound healing or increased corneal scarring from higher MICs (15, 16). Further, increased MICs from blood products may result in greater risk for development of antibiotic resistance, a growing and serious threat in veterinary and human ophthalmology (11, 17, 18). With higher MICs, bacteria may be exposed to sub-therapeutic levels of antibiotics for longer durations, a known risk factor for promoting resistance (19).

Reductions in MICs were sometimes observed in the present study, a finding that was likely related to the direct antimicrobial effect of antimicrobial peptides (e.g., lysozyme) naturally present in the serum or plasma (20). Such antimicrobial effect was more common with equine vs. canine blood products, specifically equine serum and (to a lesser degree) equine plasma and was mostly observed for *Staphylococcus pseudintermedius* and (to a lesser degree) *Pseudomonas aeruginosa*. In another study, Yates *et al* assessed the antibacterial efficacy of canine and equine autologous conditioned plasma and amniotic membrane eye drops against several ocular pathogens, including *Streptococcus canis*, *Streptococcus bovis*, *Staphylococcus hyicus*, *Staphylococcus intermedius*, *Staphylococcus schleiferi*, *Staphylococcus aureus*, *Staphylococcus warneri*, *Staphylococcus epidermidis, Pseudomonas aeruginosa*, *Bacillus cereus*, *Enterococcus faecalis*, *Corynebacterium pseudotuberculosis*, *Lactobacillus* sp., and *Escherichia coli* (21). In that experiment, amniotic membrane drops did not have antimicrobial effect against any bacterial isolates, while autologous conditioned plasma only partially inhibited the growth of a single bacterial species (*E. faecalis*). Taken together, it appears the potential antimicrobial effect may depend on various factors such as the type of blood product (e.g., serum, plasma, other), species of origin, and specific bacterial pathogen. In particular, the superior antibacterial activity of serum vs. plasma has been reported decades ago by Hirsh (1960) (22), a finding that was potentially linked to antimicrobial factors released from leukocytes and platelets that may not be present prior to clotting (i.e., plasma) (22). Further investigations into the proteomic components of blood products may help elucidate the potential compound (s) responsible for the direct antimicrobial properties. Regardless, such antimicrobial properties may help explain the lower reduction in MICs observed in the present study when growth media were supplemented with equine vs. canine blood products, despite the overall higher albumin levels in equine blood products.

Bacterial keratitis is a serious and vision-threatening condition in veterinary and human patients with a zoonotic potential (23), requiring rapid diagnosis and aggressive medical management to promote healing and minimize deleterious effects to the eye. In practice, clinicians often use topical blood products at a high frequency (i.e., up to every 1–2 h) in addition to topical antibacterial therapy. On one hand, topical blood products provide several benefits for the management of clinical patients with bacterial keratitis. First, serum and plasma contain α2-macroglobulin and α1-proteinase inhibitors that help mitigate corneal collagenolysis by reducing the activity of matrix metalloproteases and serine proteases on the ocular surface (1, 24). Second, serum and plasma contain several growth factors that can assist with corneal wound healing, such as platelet-derived growth factors, transforming growth factor beta, insulin-like growth factor, vascular endothelial growth factor, fibroblastic growth factor, and epidermic growth factor (25). On the other hand, it is important for clinicians to recognize the drawbacks of topical blood products and break the misconception that they can only be good when used topically for diseased eyes. For one, there is strong clinical evidence (i.e., placebo-controlled, randomized, masked clinical trials) that blood products do not accelerate re-epithelialization in canine patients with corneal defects (26, 27). Further, the present study shows potential interferences between blood products and antibiotics when used concurrently, often reducing the antibiotic efficacy against ocular pathogens commonly isolated from canine patients with bacterial keratitis (e.g., *Staphylococcus pseudintermedius*, *Streptococcus canis*, *Pseudomonas aeruginosa*) (11, 28).

In practice, clinicians might consider protein-free alternatives to blood products for addressing corneal collagenolysis in clinical patients, including topical N-acetylcysteine or topical ethylenediaminetetraacetic acid (EDTA) (6). If using a topical blood product, the following recommendations can be offered from the present study: (i) Prefer equine blood products as the impact on antibiotics was lesser and the impact on bacterial growth was greater with equine vs. canine blood products; (ii) Prefer serum over plasma as the impact on bacterial growth was greater and there were no differences in albumin content between the two blood products; (iii) Use fresh or frozen blood product as there were no differences in albumin content or antibacterial efficacy between the two storage conditions; and (iv) Apply the topical blood product last, at least 15 min or longer following the last topical antibiotics. For the latter, at that time, less than 5% of the drug generally remains in the tear film (29), thus minimizing the negative impact of antibiotic-albumin binding when the topical blood product is administered.

The main limitation of this study is the *in vitro* nature of the experiments. As such, the present findings cannot be directly extrapolated to clinical patients where anatomical and physiological factors also play a role in the efficacy (or lack thereof) of antibiotics against ocular pathogens. For instance, painful eyes from bacterial keratitis might have excessive reflex tearing, frequent blinking and efficient nasolacrimal drainage that result in rapid loss of topical medications (30, 31). Further studies are required in clinical patients to assess the potential impact of blood products on topical antibiotics *in vivo*. Another study limitation is the focus on two blood products from two species as results may differ with other blood product types (e.g., platelet-rich plasma) (32) or blood products originating from other species (e.g., humans) (33).

In summary, blood products modulated the MICs of ophthalmic antibiotics against common ocular pathogens from patients with bacterial keratitis. When using blood products in clinical practice, clinicians should consider equine over canine blood products (lesser impact on antimicrobial susceptibility), serum over plasma (greater antimicrobial effects), fresh or frozen, and administering the blood product ≥15 min following the last antibiotic eyedrop to minimize the amount of albumin-antibiotic binding in the tear film.

## Data availability statement

The raw data supporting the conclusions of this article will be made available by the authors, without undue reservation.

## Ethics statement

The animal study was approved by Institutional Animal Care and Use Committee at Iowa State University (protocol # 21-197). The study was conducted in accordance with the local legislation and institutional requirements.

## Author contributions

MK: Conceptualization, Data curation, Investigation, Methodology, Project administration, Supervision, Writing – original draft, Writing – review & editing. MR: Investigation, Project administration, Writing – review & editing. CS: Methodology, Writing – review & editing. DK: Methodology, Writing – review & editing. RA: Methodology, Writing – review & editing. LS: Conceptualization, Data curation, Formal analysis, Methodology, Resources, Validation, Writing – review & editing.

## Footnotes

^1^assets.thermofisher.com/TFS-Assets/MBD/Specification-Sheets/Sensititre-Plate-Layout-JOEYE2.pdf
